# Esophageal Variceal Band Ligation Interval and Number Required for the Obliteration of Varices: A Multi-center Study from Karachi, Pakistan

**DOI:** 10.7759/cureus.4993

**Published:** 2019-06-25

**Authors:** Nazish Butt, Amanullah Abbasi, M Ali Khan, Sehrish Butt, Syed Masroor Ahmad

**Affiliations:** 1 Gastroenterology, Jinnah Postgraduate Medical Centre, Karachi, PAK; 2 Internal Medicine, Dow University of Health Sciences, Karachi, PAK; 3 Biomedical and Biological Sciences, Dow University of Health Sciences, Karachi, PAK; 4 Internal Medicine, Jinnah Sindh Medical University, Karachi, PAK

**Keywords:** esophageal varices, band ligation, sessions, interval

## Abstract

Introduction

Esophageal variceal band ligation (EVBL) is the best form of treatment for variceal bleeding. The frequency of EVBL for the eradication of esophageal varices has no consensus. We evaluated the number and interval of EVBL sessions required for the obliteration of esophageal varices.

Methods

Esophagogastric varices were treated endoscopically with band ligation on initial presentation and then every after three weeks till the obliteration of the varices. Endoscopic band ligation consists of placing rubber elastic bands on large varices. Frequencies were calculated for qualitative variables and mean ± standard deviations for continuous variables.

Results

A total of 107 cases with esophagogastric varices were enrolled. Out of them, seven patients with small esophageal varices and large fundal varices were excluded. The remaining 100 with large esophageal varices had EVBL performed. The second session of EVBL was done in 46 patients with large esophageal varices. The third session of EVBL for the obliteration of esophageal varices required in 20 patients with large esophageal varices and the fourth session was required in only two patients. The total sessions required for the complete obliteration for esophageal varices were 2±1. Only one patient developed post-EVBL bleeding one week after band ligation.

Conclusion

Esophageal variceal ligation was a safe and well-tolerated procedure performed at three-week intervals in patients with large esophageal varices. On average, two to three sessions of EVBL are required for the complete obliteration of esophageal varices.

## Introduction

Esophageal varices are seen in approximately 50% of patients with portal hypertension and are a major cause of morbidity and mortality in patients with chronic liver disease [1]. Variceal bleeding occurs at a yearly rate of 5%-15%. The highest risk of first bleeding occurs in patients with large varices and advanced liver disease [2].

Esophageal variceal band ligation is the best form of management for variceal bleeding until liver transplantation or surgical shunting is unavoidably required as a result of recurrent bleeding [3]. The frequency of esophageal variceal band ligation (EVBL) for the eradication of esophageal varices has no consensus. Some authors reported a minimum of one month between banding procedures while others perform EVBL on a weekly basis [4-6]

Also, little data is available on the number of sessions required to achieve complete variceal eradication in Pakistan. Therefore, the aim of this study was to determine the number of EVBL sessions and interval required for the obliteration of esophageal varices.

## Materials and methods

The study was conducted between January 2017 and December 2018 at Ward 23, Gastroenterology section, and Medical Ward III of Jinnah Post Graduate Medical Centre, Karachi, along with Medical Unit II, Dow University of Health Sciences, Ojha Campus, Karachi. Written consent was taken from all patients. All details about the patients were kept confidential.

Consecutive cases of esophagogastric varices were treated endoscopically with band ligation and follow-up endoscopy with or without banding was done every three weeks after the initial session until the obliteration of varices was achieved. EVBL is a procedure that comprises placing rubber elastic bands on esophageal varices. The terminology and selection of variables were based on the study guidelines proposed by the Baveno VI consensus [7].

Patients with gastric varices as a cause of bleeding, hepatocellular carcinoma, and portal vein thrombosis evident on ultrasonography, parenteral drug addiction, and current alcohol abuse were excluded from the study. Data were entered and analyzed by using SPSS version 21 (IBM Corp., Armonk, NY, US). Frequencies were calculated for qualitative variables and mean ± standard deviations for continuous variables.

## Results

Of the 107 patients with esophagogastric varices, seven with small esophageal varices and large fundal varices were excluded. Out of the remaining 100 patients, 69 were male and 31 were female. Age ranged from 17 to 80 years old, with an average of 49±11 years. The baseline characteristics of the patients are shown in Table 1.

**Table 1:** Demographic and baseline characteristics. HCV: Hepatitis C virus; HBV: Hepatitis B virus; HDV: Hepatitis D virus; NBNC: Neither hepatitis B nor hepatitis C; CTP: Child-Turcotte-Pugh; MELD: Model for end-stage liver disease

Parameters	N=100 (%)
Age (Mean±Standard Deviation)	49±11
Sex	
Male	69(69%)
Female	31(31%)
Diagnosis	
HCV	77(77%)
HBV	12(12%)
HBV+HCV	04(4%)
HBV+HDV	01(1%)
NBNC	06(06%)
CTP Score	
A	16(16%)
B	59(59%)
C	25(25%)
Alpha Fetoprotein (Mean ± Standard Deviation)	30.7±___226
MELD Score (Median)	12

All patients with large esophageal varices had EVBL performed. The second session of EVBL was required in 46 patients (46%) for the obliteration of varices. The third session of EVBL for the obliteration of esophageal varices was required in 20 (20%) patients. A fourth session was required in only two (2%). A median of two sessions was required for the complete obliteration of esophageal varices. Only one patient developed post-EVBL bleeding one week after band ligation. The results are summarized in Table 2.

**Table 2:** Sessions required for variceal obliteration

(N=100)	Number of patient with obliterated varices (%)
After First session	32 (33%)
After Second session	46 (46%)
After Third Session	20 (20%)
After Fourth Session	2 (2%)
Median	2 sessions

## Discussion

This study evaluated the interval and number of sessions of EVBL required for the obliteration of esophageal varices. EVBL is currently the endoscopic treatment of choice for esophageal varices. Previous studies have suggested that sessions of EVBL should be continued until the achievement of complete obliteration of varices. Varices are usually considered eradicated when they have either vanished or cannot be grasped and banded by the ligator [8].

We observed that age and gender distribution was similar as reported in other studies. However, the cause of cirrhosis and portal hypertension was overwhelmingly dominated by hepatitis C virus (HCV)-related liver cirrhosis; 77% in our study as compared to 39% in other studies. This can be explained by the high prevalence of HCV in Pakistan. Most of our patients belonged to Child-Pugh class B or C (84%), whereas other literature subsets had a dominance of Child A patients (82%).

Within this region, a study to evaluate the number of sessions required for the obliteration of varices has not been conducted. The same can be said about an agreement on the interval between each session, the need for which has been highlighted in recent articles. In our clinical pathway, sessions were arranged at three-week intervals to achieve variceal eradication. A study by Yoshida et al. advocated that EVBL be performed every two months and that this was better than EVBL performed once every two weeks regarding overall rates of variceal recurrence and additional treatment. It also showed a higher total eradication rate [9].

Another study conducted in Morocco with long-term follow-up achieved the complete obliteration of esophageal varices in one to eight sessions (mean 3) conducted over three to 28 weeks (mean 14 weeks) [8]. The reported rebleeding rate of patients receiving endoscopic therapy could only be significantly reduced in those who achieve variceal obliteration within a short period. EVBL performed at an interval of two months for the prevention of variceal rebleeding may, therefore, be inappropriate, which is in accordance with our set interval of three weeks.

Another minor but relevant reason for a three-week interval was the compliance of patients, who tend to be lost to follow-up with intervals greater than one month, and are unwilling to undergo the process at weekly intervals due to discomfort and expenditure. Obliteration was achieved in 32% of our patients after a single session of EVBL. The reason for the achievement of obliteration after a single session of EVBL was the spontaneous decrease in the hepatic venous pressure gradient, which occurred in around 30% of patients treated with EVBL [10-11].

Patients with such a natural hemodynamic response required fewer sessions of EVBL until variceal obliteration and have a higher rate of variceal eradication than patients treated with endoscopic methods who have no spontaneous response [11]. Nearly a third of patients responding to a single session of EVBL suggests a similar response in our study; 46% required two sessions and only 22% needed three or more sessions.

EVBL is a life-saving procedure and significantly reduced the frequency of variceal rebleeding, morbidity, and mortality. The main limitation of our study was three limited sample size and follow-up. Data need further evaluation on a larger scale, with a longer follow-up duration at multiple centers to establish consensus.

## Conclusions

EVBL was safe and well-tolerated in patients with large esophageal varices. The three-week interval in between sessions ensured follow-up, compliance, and the eventual obliteration of varices. On average, two to three sessions of EVBL were required for the complete obliteration of esophageal varices.
